# Supplementary figures and images for: The Genetic Structure of Cape Verdean Population Revealed by Y-Chromosome STRs
Source: Genes (Basel). 2025 Aug 25;16(9):999. doi: 10.3390/genes16090999 (PMC12469677; doi:10.3390/genes16090999)

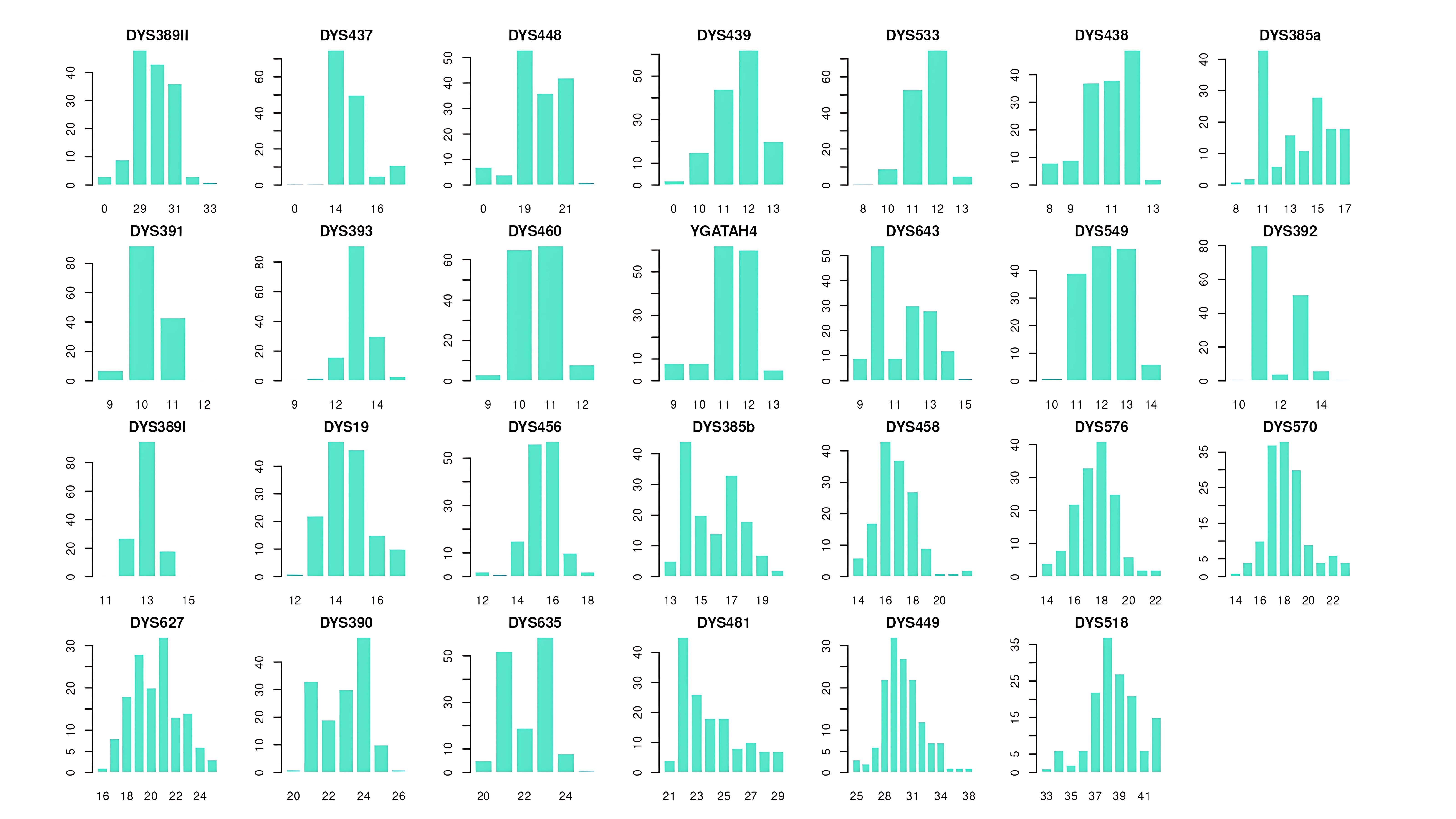

Supplement: Supplementary file 1 [file genes-16-00999-s001.zip › Figure S1.png]
